# How and why does the disease progress? A qualitative investigation of the transition into long-standing anorexia nervosa

**DOI:** 10.1186/s40337-021-00458-w

**Published:** 2021-08-17

**Authors:** Catherine Broomfield, Paul Rhodes, Stephen Touyz

**Affiliations:** 1 School of Psychology, Griffith Taylor Building, The University of Sydney, Sydney, NSW 2006 Australia; 2 InsideOut Institute, Charles Perkins Centre, The University of Sydney, Sydney, NSW 2006 Australia

**Keywords:** Anorexia nervosa, Long-standing, Transition, Progression, Trauma, Functionality, Identity, Treatment

## Abstract

**Objective:**

Despite an increased interest in understanding characteristics of long-standing anorexia nervosa (AN), there is a lack of knowledge into the processes that occur with the development and maintenance of the disease. This has impeded the development of novel treatment approaches that may prove more effective than traditional medical models of therapy. To improve the prognosis of these long-standing presentations, an understanding as to how and why the AN disease progresses is required. It was therefore the aim of the current study to investigate the transition of AN from earlier to later stages.

**Method:**

The study adopted a narrative inquiry approach and a total of 11 women with long-standing AN participated in an interview. The newly developed photovoice method assisted in data collection with typologies of chronic illness facilitating the emergence of salient themes.

**Results:**

The qualitative analysis resulted in the identification of five themes: (a) transition, (b) trauma, (c) functionality, (d) identity, and (e) failure of current models of treatment.

**Conclusions:**

Together with identifying key themes, the study provides insight into some possible reasons why current treatment models are failing to promote recovery. Future research examining the effectiveness of treatment that targets underlying causes and maintaining factors of the illness are suggested. Additional education for health professionals is also recommended in order to reduce the trauma that is currently being experienced by some patients with a long-standing illness.

## Plain English summary

Anorexia nervosa (AN) is a complex illness that has the potential to develop into a long-term presentation. When this occurs, the best way of treating this stage of the disease is currently being debated with new models of care being investigated and compared to more traditional approaches to treatment. One of the difficulties in developing more effective treatment is the lack of understanding into how and why the AN illness transitions from an earlier to later stage. It was the aim of the current study to discover the changes that occur as the AN illness progresses. A total of 11 women were interviewed to discuss their experience of AN as it progressed into a long-term illness. It was discovered that across participants, five themes emerged: (a) transition, (b) trauma, (c) functionality, (d) identity, and (e) failure of current models of treatment. Possible reasons as to why current treatment approaches are not working for long-term AN are discussed. Recommendations are made for improving education for health professionals so as to reduce the trauma that is currently being experienced by some individuals with a long-standing illness.

## Background

Anorexia nervosa (AN) is a complex illness that manifests in various ways making the eating disorder difficult to treat and the recovery process slow [1, 2]. Interest into later stages of the illness has corresponded with an increased focus on understanding these presentations in order to ascertain more effective treatment options [3–5]. One barrier to developing effective treatment includes limited information regarding how and why the disease progresses. This lack of knowledge limits the ability of researchers and health professionals to understand why AN can become so resistant and what aspects of the disease are not being adequately addressed in current treatment models.

With an estimated twenty per cent of patients developing a longer-term presentation [6], quantitative [7–9] and qualitative [10, 11] research methods have been utilised in order to better understand the characteristics of long-standing AN. Despite these efforts, there is still a limited understanding of this complex stage of the illness, especially in relation to how and why this presentation develops. In a qualitative study investigating the personal meaning of symptoms and treatment approaches, Stockford et al. [12] identified that a lack of early identification and implementation of interventions was a theme that emerged from a severe and enduring AN sample. A contribution to the development of a protracted course of AN may be the absence of parental support and lengthy waiting lists at treatment facilities [12]. Social support systems also emerged as part of the 15 themes identified by Robinson et al. [13] whilst investigating the management of patients with an AN illness duration of 20 years. Based on the findings from this study [13], psychological and social functioning were determined to be the most negatively impacted domains by this long-standing eating disorder.

In a study investigating the recovery of patients with severe and enduring AN, Dawson et al. [14] described motivation, support, self-efficacy and hope to be fundamental aspects in treating these presentations. This was in contrast to previous methods that resulted in unsuccessful treatment attempts with participants reporting an external locus of control that prevented autonomy over their own recovery [14]. Although research efforts have attempted to understand characteristics of the illness [9, 12, 13], there has been a greater overall focus to date on understanding the development of the acute stage of AN [15–17] and recovery from the illness [14, 18, 19]. What is missing in the field is an understanding as to the development of the later stage of the illness—as well as its maintenance over a long period of time—which is essential when developing more effective and targeted treatment options for these presentations.

It was crucial in the current study to adopt a qualitative research approach given underlying experiences can be missed when using quantitative methods [20, 21]. This approach would allow first-hand knowledge to be acquired as to these experiences from the perspective of affected individuals [22]. Narrative inquiry as a qualitative research method captures and analyses life stories, allowing for rich detail to be gathered on topics that are human centred and complex [23]. By illuminating the lived experience, this technique facilitated the investigation as to how and why the illness perseveres. It was the aim of the current study to track processes that occur as the AN illness progresses.

## Method

### Participants

Participants were required to meet the following self-reported criteria: (a) be at least 18 years of age; (b) currently or previously have met the Diagnostic and Statistical Manual of Mental Disorders, fifth edition (DSM-5) criteria for AN [24]; and (c) currently or previously have experienced the AN illness for a duration of seven or more years [25]. The decision to recruit participants using illness duration was based on this criterion being the most frequently cited definition in the literature for the later stage of AN as indicated in a systematic review by Broomfield et al. [25]. By adopting the same inclusion criteria, the findings in the current study were comparable to other research that has been dedicated to this subpopulation. Recruitment took place through snowball sampling (method of detecting participants through social networks) [26], social media and clinician recommendation in Sydney, Australia. Participants were assessed and included if they consented and met the inclusion criteria. This study was approved by the Human Research Ethics Committee at the University of Sydney.

## Research design, data collection and procedure

The research design included a narrative inquiry approach with the use of the newly developed photovoice method. Narrative inquiry is advantageous when researching a process that occurs over a period of time, which is particularly relevant when considering illness transition that can span many years [27]. For the current study, personal processes and the meaning behind lived experiences was prioritised in the construction of narratives.

Photovoice as a method allows participants to communicate aspects of their lives through the use of photographs [28]. This facilitates the development of an affective response and allows the story to become real for both the participant and the researcher [28, 29]. Shaped by feminist theory, this method of data collection is guided by participants allowing for a deep exploration of the often taken-for-granted lived experience of women [30, 31]. Participants were instructed to provide up to 10 photographs related to their lived experience with AN. This allowed participants to symbolise the most salient aspects of their journey, with experiences outside of these photographs discussed in order to extrapolate a timeline of events. Along with the photographs, participants were asked to record: (1) what was in each photograph, (2) what each photograph meant to the participant and (3) why each photograph was taken [32].

Limitations of the photovoice method include ethical considerations regarding consent of individuals appearing in photographs, as well as the possibility of meaning in photographs being misconstrued [33]. To overcome these limitations, participants were instructed to only include individuals in photographs who had provided written consent with consent forms provided to participants at the beginning of the study (consent forms were approved by the Human Research Ethics Committee at the University of Sydney). Photographs were explored in interviews to avoid the researcher misinterpreting the meaning of images. Data collection took place through face-to-face interviews or via Skype and was audio-recorded. The first two thirds of the interview was focused on an examination of the selected photographs with the final third used to extrapolate and explore potential underlying meanings. Prompting for further details was at times required in order to co-produce a descriptive timeline of the participant’s experience (see Table 1 for Interview Questions).

**Table 1:** Interview questions

Can you tell me what your AN means to you?	
How do you experience AN?	
How long have you been experiencing AN?	
Were there any time periods during your life where the experience of AN was particularly consuming, important or had a major impact on your life?	
Were there any time periods where the opposite was found, in that the illness had little impact on you and your life?	
Was there a specific time where you noticed or realised that the AN you were experiencing was going to be enduring?	
Can you tell me the journey you have had with AN?	
Is there anything that you would like health professionals and the research community to know about your experience of AN?	

*NoteAN* Anorexia Nervosa

The interview was then transcribed verbatim so as to capture and preserve the “voice” of participants by using their own words as much as possible. Similar to the method used by Dawson et al. [14], identified processes were placed in a temporal order to examine commonalities across narratives. The interview took approximately two hours to complete with additional time for member checking (a process whereby the analysis is checked by participants with any requested changes made to increase precision) [22].

### Data analysis

After member checking was complete, an inductive analytic process took place whereby data relating to the experiences of the illness were studied for emerging themes [34]. Typologies proposed by medical sociologist Arthur Frank [27, 35] were used as a guide to help make meaning out of the emerging concepts, with this model allowing for chronic illness to be represented through a variety of lenses. This included stories of chaos, quest, restitution and illness-as-normality, with these typologies present to varying degrees across the narratives of participants. The type of story told through each narrative was found to be changeable, with some stages of the AN illness representing one lens, which would later transform to another lens with the progression of the disorder. Through an exploration of these four lenses [27, 35] a greater understanding of the ways in which AN transitioned in the experiences told by participants became clear. After each narrative was investigated using these typologies as a guide [27, 35], commonalities across the experience of long-standing AN became salient. Links were established between the raw data and the aim of this research (i.e., to determine the transition of AN from earlier to later stages of the illness) [36], which further highlighted similarities across narratives. Overarching themes were discovered with evidence of these higher-order conceptualisations investigated in the data until a “best fit” model was reached [37]. Inductive thematic saturation was adopted in the current study, a process whereby no new information can be generated by additional data as demonstrated by no new themes emerging from the analysis [38].

### Methodological rigor

The methodology was evaluated based on criteria relating to credibility, fittingness and auditability [39]. Credibility was maintained by using direct quotes when possible to construct narratives. To ensure accuracy, participants were offered the opportunity to member check their narratives. Participant characteristics were described in order to facilitate an understanding of fittingness. Auditability was demonstrated by a thorough audit trail kept in order to ensure analytic transparency [39]. To reduce bias, narratives were cross-coded by two authors with any disagreements resolved through discussion.

## Results

A total of 11 women participated in the study. As required, all participants self-reported meeting the DSM-5 criteria at some point during their seven or more year experience with AN [24, 25]. For participant demographics, see Table 2.

**Table 2:** Participant demographics

Characteristic	*n* (proportion)	Mean (SD; years)	Range (years)
Age		41.6 (11.5)	29–66
Duration of illness^a^		26.2 (13.2)	7–53
Stage of illness			
Currently ill	6 (54.55%)	–	–
Recovering	3 (27.27%)	–	–
Recovered^b^	2 (18.18%)	–	–
AN subtype			
Restricting	9 (81.82%)	–	–
Binge-eating/purging	2 (18.18%)	–	–
Employment status			
Unemployed	4 (36.36%)	–	–
Casual	2 (18.18%)	–	–
Part-time	1 (9.09%)	–	–
Full-time	4 (36.36%)	–	–
Marital status			
Single	8 (72.73%)	–	–
De-facto/married	3 (27.27%)	–	–
Children			
No	8 (72.73%)	–	–
Yes	3 (27.27%)	–	–
Language spoken at home			
English	11 (100%)	–	–

*NoteAN* Anorexia Nervosa. BMI was not collected as irrelevant for the current study

^a^Each participant provided their own indication of when their illness began. The decision to have women decide the illness starting point was in line with the current research method of working with lived-experience participants who are regarded as the expert on their experience. The majority of participants regarded the starting point of their illness to be when they themselves first noticed symptoms of AN (*n* = 10), with some of these women (*n* = 2) also receiving a diagnosis by a health professional the same year symptoms began. The remaining participant (*n* = 1) regarded the illness starting point to be when family and friends first noticed symptoms

^b^Although classified as recovered, participants (*n* = 2) still experienced cognitive symptoms related to AN

Photographs provided by participants varied in content. Images consisted of significant individuals, triggers of illness, physical activities and hobbies.

### Themes

There were a total of five themes that emerged from the data during analysis: (a) transition, (b) trauma, (c) functionality, (d) identity, and (e) the failure of current treatment models.

#### Theme 1: Transition

All participants (*n* = 11) described transitional periods throughout their illness. The most common transition related to the progression from an acute to a later stage of the disease with the manner of in which this transition occurred varying across experiences. Some of the women (*n* = 8) experienced distinct shifts when the illness progressed into long-standing AN with the remaining women (*n* = 3) experiencing a more linear progression with no distinct turning points.

When a distinct turning point was experienced, some of the women (*n* = 5) identified this to have occurred when their life had become completely consumed by the illness:

“When I was younger, I could have periods of time where I was a bit better or, you know, it wasn’t 24 hours a day. I wasn’t dreaming about it, you know, but then when I got older, there was no, nothing outside the eating disorder. Nothing escaped that filter; it was like it was enclosed in your head. There was nothing else.” – Participant 3

This consumption of their life by the illness was often paired with an overwhelming sense of grief when there was a realisation that recovery might be harder to achieve than originally hoped:

“It was horrible in that sense of grief…that’s what I would, would call ‘severe and enduring’ as when it’s become such a huge part of your life, it’s become more of your life than your life itself and so, that’s why I think that it’s harder to recover from…” – Participant 9

For some women (*n* = 2), the transition into long-standing AN was paired with a life-changing experience that brought a sense of acceptance of the illness, which propelled them towards recovery. This included being confronted by life-threatening consequences if failing to address the disorder (*n* = 1) and an unsuccessful suicide attempt (*n* = 1). In another experience (*n* = 1), the turning point came after all available treatment models were attempted with little success:

“At that moment it was huge grief, huge grief, uhmm, which I still feel sometimes right, because part of, part of what letting go of the idea of getting better and recovering means is letting go of all the things I had hoped to do and hoped to be able to do with ease. Uhmm and as you can see that still makes me sad, uhmm cause there are always going to be things that are inaccessible to me and that’s both okay and tragic.” – Participant 5

Other transitional periods included turning points towards recovery (*n* = 5), the ability to contribute towards society through work (*n* = 3), or in contrast, being unable to continue work as a result of health complications (*n* = 4). Entering into new roles was another transition for some participants including becoming a mother (*n* = 3). These experiences required participants to shift their relationship with the eating disorder in order to meet the demands of new roles, which at times involved recovery, periods of remission or continuing along the trajectory of their long-standing eating disorder.

#### Theme 2: Trauma

Trauma was present across narratives during different times of the illness with all participants (*n* = 11) reporting to experience some form of trauma that pre-dated the development of the eating disorder. In most cases (*n* = 8), the trauma immediately preceded the development of symptoms relating to AN. The more common forms of trauma developed from witnessing abuse or tension in the family home (*n* = 3), being sexually abused by a family friend (*n* = 2) or date (*n* = 1) and being physically abused by a parent (*n* = 1) or sibling (*n* = 1):

“I think it was just a loaded gun…probably the main trigger for my anorexia was the abuse.” – Participant 6

“I found very complicated ways of trying to manage what had happened [rape] and just made things worse… in that sense my life did get out of control, because I was trying to undo something that had happened by doing other things, that ended up making it worse…” – Participant 8

Disconnected relationships with family (*n* = 5), friends (*n* = 2) or both (*n* = 2) was reported as traumatic with the ensuing isolation preventing other people from intervening in the progression of the illness. In some circumstances (*n* = 5), this left AN to go undetected for long periods of time.

The embodiment of trauma occurred for many of the women (*n* = 7) after feeling pressure from family (*n* = 1), peers (*n* = 1) or both (*n* = 1) to lose weight. Other women (*n* = 4) experienced a sense of competition with themselves.

“…I think it [AN] had a lot to do with being really traumatised in my body.” – Participant 9

When other people communicated displeasure over the participant’s body (*n* = 3), the impact of their criticism manifested in numerous ways. Adopting dieting techniques from parents (*n* = 1) or being placed on a diet (*n* = 2) were some of the ways participants began their difficult relationship with food, which for one woman occurred as early as four years of age.

#### Theme 3: Functionality

The theme of functionality related to the way in which the illness served a purpose in the lives of the women, which ultimately made recovery difficult to achieve. Although there were multiple reasons for the illness having a prolonged trajectory, all of the women (*n* = 11) described the fundamental purpose of AN as a way to regain a sense of control over certain aspects of their lives. For most participants (*n* = 9), this was in relation to the body itself with a loss of control through physical development and puberty (*n* = 3), becoming victimised through sexual (*n* = 3) or physical abuse (*n* = 2) and the loss of bodily functions through the symptoms of another chronic health condition (*n* = 1).

As AN transitioned into a later stage, the function of the illness also transitioned for most participants (*n* = 8). With the illness initially functioning as a way to regain control, AN eventually became a way to manage subsequent distressing emotions such as grief (*n* = 3), maintaining an idealised body shape (*n* = 2), forming part of the individual’s identity (*n* = 2) and encompassing a perspective for experiencing the world (*n* = 1).

The use of the illness to avoid experiencing distressing emotions (*n* = 10) acted as an original function of AN for some participants (*n* = 7) and developed as the illness progressed for other participants (*n* = 3). For individuals who were experiencing symptoms of post-traumatic stress as a result of sexual (*n* = 3) or physical abuse (*n* = 2), AN was used as a method to drown out distressing emotions and focus attention on pain inflicted upon themselves:

“I wanted the physical pain as opposed to the emotional because that made sense. I could recognise that, that’s what was wrong whereas emotionally it was just too, you know, too hard to identify it.” – Participant 9

“…nothing can harm you because of the way you’re harming yourself, basically.” – Participant 1

Other incidents that caused social isolation (*n* = 3) and stress (*n* = 2) were often paired with difficult emotions that some of the women felt incapable of managing without the illness:

“…as long as I’m in the anorexic range I can cope with life. The second I’m not, I can’t.” – Participant 6

By removing complications in life and assisting in the management of distressing emotions, AN offered participants (*n* = 11) safety:

“Anorexia offered this really clean, pure, serene, space that really contrasted to all that messy, ugly, nasty, out of control stuff…it’s a safer place to be.” – Participant 8

#### Theme 4: Identity

Although identity was a theme that emerged throughout most of the experiences (*n* = 10), there was a clear division in the way that women identified with the illness. There was an even split in the experiences involving identity with half of the women (*n* = 5) identifying with the illness and the other half (*n* = 5) rejecting a personal interconnection. The degree of illness identity also changed as the disease progressed. Some of the women (*n* = 5) who previously identified with AN re-formed their identity during recovery (*n* = 3) or shifted their perspective away from the problematic features of the disease (*n* = 2):

“I think part of the reason that I maybe still identify with the term [AN] is I tend not think of it as an illness.” – Participant 8

Given the typical development of AN occurs during adolescence and then progresses into adulthood for long-standing presentations, women reported a consistent and dependable aspect of their lives to have become the disease:

“It [AN] just gets into everything and particularly over time...it just seeps into every part of your life…” – Participant 8

At times, the illness needed to be grieved in order to reach a point of recovery (*n* = 1):

“…why I think a lot of people with ‘severe and enduring’ have ‘severe and enduring’, cause the acceptance of grief and losing a part of yourself, what’s been a part of yourself for so long becomes even harder… It’s not that I loved my eating disorder but it had become a part of me and my life, so letting go of something, that was so now engrained was a huge thing uhmm, for me it was like losing a child.” – Participant 9

The reasons for women rejecting an identification with AN (*n* = 5), included the illness interfering with their values (*n* = 2), violating professional (*n* = 1) or personal (*n* = 1) goals as well as regarding their illness as not being severe enough in their life to associate with personally (*n* = 1).

“I was very, very aware of the two selves that were happening, you know, the crazy self and the sane self that was watching, which sounds awful doesn’t it? Uhmm and it was a bit scary at the time actually of being driven by something you didn’t understand and you didn’t think was necessarily part of you…” – Participant 7

#### Theme 5: Failure of current models of treatment

The failure of current models of treatment was found in the experience of almost all women (*n* = 10), except for one participant who avoided the health care system. There were commonalities identified across the majority of participants (*n* = 10), which included a lack of success in long-term recovery following the attempt of evidence-based treatment approaches offered between 1960 and 2018. This often involved attempting different models of treatment that were available during the time of their illness:

“I tried everything, all the different three letter acronym-types of therapy, uhmm, different day programs, bunch of clinical trials, uhmm, yeah and in-patient was the only thing I hadn’t done and I did that, and it didn’t help.” – Participant 5

The second most common failure of treatment included a general lack of understanding about long-standing AN from treating staff (*n* = 8). Protocols used in treatment facilities revealed a lack of awareness by clinicians of fundamental aspects of the illness. By denying a patient their sense of autonomy through the process of treatment, attempts at recovery were often ineffective and left the participant feeling more traumatised than before they were admitted into therapy. For some participants (*n* = 5), this involved treating staff using threats in order to persuade patients to increase their food intake, a method reported by the current sample to have occurred between 1980 and 2016. This included threats to ban visits from family and friends (*n* = 3), withholding hospital leave (*n* = 1), phone calls (*n* = 1), personal clothing items (*n* = 1) and blankets (*n* = 1) if patients did not comply:

“In the in-patient it was very much that idea of, ‘If you eat your food you can have visitors, if you eat your food you can have a blanket, if you don’t you can’t’. Uhmm, again, I’m not really entirely sure what they were hoping that would achieve, uhmm it didn’t really achieve very much other than I got very cold and very miserable.” – Participant 8

Treatment facilities also threatened patients with involuntary tube feeding (*n* = 2; between 1998 and 2018), scheduling (*n* = 1; between 2000 and 2010) and being tied to a bed (*n* = 1; between 2008 and 2018) if they did not comply with treatment guidelines. For some participants (*n* = 4) who presented to facilities, there was also rejection by staff. Reasons for rejection included their illness being too complex (*n* = 2; between 1998 and 2018), not severe enough (*n* = 1; between 2000 and –2010) and the individuals being too disengaged/avoidant during previous admissions (*n* = 1; between 2008 and 2018):

“…I know there are like hundreds of people who fall through the cracks for being too thin or self-harming…like the system is so broken in that sense. Uhmm but that is such an awful feeling to be asking people to help you and them saying, ‘Well, prove to us why we should.’” – Participant 5

Abuse and adverse experiences were also reported (*n* = 3), which included treating staff sexually assaulting patients (*n* = 1; between 2000 and 2010), force-feeding and incorrectly placing feeding tubes (*n* = 1; between 2008 and 2018) as well as unsupported treatment methods for AN being recommended, such as a lobotomy and deep sleep therapy (*n* = 1; between 1960 and 1970):

“I think the treatments probably done more harm than good you know, I need to recover from the treatment more than I need to recover from the disorder. Uhmm yeah if you ask me, you know, the most damaging thing in my life, I’m not going to say anorexia, I’m going to say treatment for anorexia” – Participant 11

## Discussion

Five themes were identified whilst investigating the progression of AN. The theme of transition was a common experience and a distinct turning point was described by most participants during their development of long-standing AN. Given this was often described in the context of a realisation that most of their lives had become permeated by the disease, incorporating this indicator into an assessment tool could assist in identifying these presentations. Additionally, targeting the associated grief that was often paired with this transition may be important to address during therapy.

The finding that trauma pre-dated the development of AN was consistent with previous research that has identified trauma as a risk factor [40, 41]. When trauma^1^ was described to result from the breakdown of close relationships, it is worthwhile to determine this influence on a prolonged illness trajectory through further research. With early intervention shown to improve outcomes from AN [42], limited social support during the onset of the illness may have prevented recognition of AN symptoms and reduced any corresponding encouragement by social networks to seek treatment during earlier stages of the disease. A lack of early identification and implementation of interventions was a theme identified by Stockford et al. [12] in their severe and enduring AN sample, which was similarly suggested to perhaps be contributed to by the absence of parental support during the early stages of the illness. Although not all participants in the current study described this form of trauma, further investigation into the effect of a disrupted social network on the likelihood of transitioning into long-standing AN is warranted.

With all participants in the current study describing a loss of control as contributing towards the aetiology of their illness, these findings are consistent with earlier models of AN, which suggested that the disease developed as a method to compensate for loss of control [44]. A unique finding in the current study was how the function of AN changed over time for some participants. In terms of improving current treatment methods, ensuring that underlying causes of the illness are addressed may be important given the potential link between functionality and maintenance of the disease. Targeting symptoms alone may be insufficient for patients who have used AN as a method for coping with challenges over a substantial period of time. It is therefore important that the functionality of the disease is continuously assessed during treatment.

Stockford et al. [12] described identity to be a major theme in their sample of severe and enduring AN, which is consistent with the findings of the current study. It is recommended that assessments include an investigation into the role of the illness in the identity formation of patients, and that this be addressed in treatment. For patients who identify with AN, it may be beneficial for therapy to assist in the formation of new identities outside of the illness in order to provide hope for a future free of the disorder. For patients who deny AN as forming part of their identity, this variable could potentially be used as a tool in therapy. Utilising strategies such as cognitive challenging may promote recovery by highlighting the incongruence of the illness from how the patient identifies themselves as an individual.

The ineffectiveness of current treatment models was not surprising given the lack of knowledge of long-standing AN and the corresponding limited evidence-based treatments available. However, a significant finding to have emerged from this research was the participants’ report of coercive methods in some treatment facilities that left them feeling miserable and at times, stripped of their basic human rights. This may relate to the findings by Dawson et al. [14] with participants identifying unhelpful treatment to include ‘scare tactics’ and punishments as forms of motivation. Perhaps this was similar to the coercion described by participants in the current study who reported the ineffectiveness of these methods in facilitating recovery. This may be the reason why some individuals experiencing AN disengage from services [45].

Another surprising discovery was that help-seeking individuals were occasionally turned away from treatment facilities. As previously mentioned, early intervention has been found to be a predictor of better outcomes for patients [42]. This raises concerns as to whether this finding may be a contributing factor in the persistence of some cases of AN. Further research is essential in determining why health professionals are denying treatment to some patients, as is better education for staff on the potential consequences. Furthermore, the effectiveness of traditional ‘therapeutic’ strategies outlined in the current study and described in the findings of Dawson et al. [14], such as withholding personal items as forms of punishment, need to be evaluated against more novel treatment options.

A more recently applied model to presentations that persist into the later stage of AN is recovery-based models. The basis of recovery-based models is a shift in the focus of treatment from symptom elimination to improving quality of life and general wellbeing [46]. Calugi et al. [47] recommend adopting a recovery-based approach when treating patients with severe and enduring AN with findings from a longitudinal outcome study suggesting this is well tolerated and a viable option for individuals experiencing this illness. Although it was beyond the scope of the current paper to investigate whether recovery-based models prove more effective in treatment than traditional medical models, it is hoped the current findings will inspire research into discovering what approaches are most effective for long-standing AN. The authors recommend the use of mixed research methods when continuing to investigate transitions between the stages of AN with prospective research particularly beneficial for studying the development of illness over time. Furthermore, a comparison between the processes that occur in the long-standing illness with individuals who recover from AN may provide a greater understanding of the factors that differentiate these experiences from those with a better outcome.

The current study had several limitations including being unable to confirm causality with additional research required for this purpose. The convenience sampling method of the snowball technique may have biased recruitment [26]. With this being a retrospective study requiring participants to recall details from early childhood as well as at times when they were severely unwell, it is possible that there may have been recall bias, limiting the accuracy of findings. Additionally, a limitation of all qualitative research particularly from a narrative inquiry framework is the inevitability of some interpretive efforts. Although the intention is to extract meaning made by participants, narrative research involves a degree of interpretation by the researcher and an understanding of the participant differently to how they may perceive themselves [48]. To address this limitation, member checking was offered but only optional with some participants completing this process (*n* = 6), others returning incomplete edits (*n* = 3) or requesting to not be involved (*n* = 2). Although this may have affected the accuracy and validity of the data, it was important to allow participants autonomy over this process. Despite keeping recruitment open to all genders, only females participated, which limited the generalizability of these findings to other genders. Additionally, the recruitment of participants based on the inclusion criterion of illness duration may be argued as a limitation in terms of the theme that emerged in the data analysis of failure of current models of treatment. Individuals were required to have a long-term illness to participate and so the emergence of this theme was not an altogether surprising finding. However, this theme was important and directly addressed the aim of the current study to investigate how and why the AN illness transitions from an early to late stage. Furthermore, there was initial interest expressed in the current study by 26 individuals, with 15 people deciding against participation. This may be argued to reflect a potential bias in recruitment with the final sample perhaps only including individuals who had more time available to participate.

A narrative inquiry approach strengthened the findings in the current study by facilitating an understanding of underlying elements of the stories told by participants, which may have otherwise remained undiscovered [49]. Another strength was achieving thematic saturation, which was identified during data analysis after recruiting a particularly large sample of participants. Previous qualitative research on long-term AN has featured up to eight participants [12–14]. Accordingly, a strength of the current study could be argued to be that it achieved one of the largest sample sizes for this topic, which was required for this research to achieve thematic saturation. Additional strengths included recruiting individuals with large variability in duration of illness and at different points in their experience of long-standing AN, which meant that the findings encapsulated the variability of this disease as it manifests over time.

## Conclusion

It was the aim of the current study to provide a greater understanding of the processes that occur when AN progresses from earlier to later stages. Along with identifying the key themes of transition, trauma, functionality and identity, this research provides insight into how current models of treatment have failed these individuals. In terms of the practical implications, findings from the current study suggest that periods of transition occur along the trajectory of these long-standing presentations with the function of the illness likely to change throughout the experience. Trauma may be a common experience with disrupted social networks potentially acting as a barrier to early interventions with further research required to confirm the existence of a relationship. Identity may be an important aspect to consider when managing patients with these presentations, and potentially provide a technique for use in therapy based on the degree of association between the individual and their illness. The finding of currently ineffective and in some circumstances, harmful treatment approaches, requires further investigation. It is imperative that practices involving the mistreatment and abuse of some of these patients disguised under forms of ‘treatment’ are abolished. There is a need to employ both qualitative and quantitative methods when exploring more effective treatment approaches.

## Data Availability

Due to privacy and ethical considerations relating to maintaining the confidentiality of participants, data cannot be made available.

## Abbreviations

AN: Anorexia Nervosa
DSM-5: Diagnostic and Statistical Manual of Mental Disorders, Fifth Edition [24]

## Footnotes

1 In the current study, the definition of trauma by Herman [43] was adopted, which describes psychological trauma as the experience of powerlessness that disrupts an individual’s sense of control, connection and meaning.
